# Novel WiFi/MEMS Integrated Indoor Navigation System Based on Two-Stage EKF

**DOI:** 10.3390/mi10030198

**Published:** 2019-03-20

**Authors:** Yi Cui, Yongbo Zhang, Yuliang Huang, Zhihua Wang, Huimin Fu

**Affiliations:** Research Center of Small Sample Technology, Beihang University, Beijing 100191, China; cuiyi@buaa.edu.cn (Y.C.); huangyuliang2017@buaa.edu.cn (Y.H.); 09691@buaa.edu.cn (Z.W.); 12051159@buaa.edu.cn (H.F.)

**Keywords:** indoor navigation, MEMS sensors, WiFi, extended Kalman filter, WKNN

## Abstract

Indoor navigation has been developing rapidly over the last few years. However, it still faces a number of challenges and practical issues. This paper proposes a novel WiFi/MEMS integration structure for indoor navigation. The two-stage structure uses the extended Kalman filter (EKF) to fuse the information from WiFi/MEMS sensors and contains attitude-determination EKF and position-tracking EKF. In the WiFi part, a partition solution called “moving partition” is originally proposed in this paper. This solution significantly reduces the computation time and enhances the performance of the traditional Weighted K-Nearest Neighbors (WKNN) method. Furthermore, the direction measurement is generated utilizing WiFi positioning results, and a “turn detection” is implemented to guarantee the effectiveness. The navigation performance of the presented integration structure has been verified through indoor experiments. The test results indicate that the proposed WiFi/MEMS solution works well. The root mean square (RMS) position error of WiFi/MEMS is 0.7926 m, which is an improvement of 20.59% and 36.60% when compared to MEMS and WiFi alone. Besides, the proposed algorithm still performs well with very few access points (AP) available and its stability has been proven.

## 1. Introduction

In recent years, location-based services (LBS) have become increasingly important. Location-based systems are required in various fields such as mobile commerce, parcel or vehicle tracking, discovering the nearest shops or restaurants, and social networking [1]. In LBS, positioning is one of the key issues to be addressed [2]. On the other hand, researches have shown that people spend about 70% of their time indoors [3]. The demand for indoor navigation is increasing rapidly in a number of applications. Therefore, indoor navigation has gained plenty of attention from companies and researchers.

While Global Navigation Satellite Systems (GNSS) based outdoor positioning and navigation has achieved great advances and high accuracy in the past few decades, indoor navigation still remains an unsolved problem [4,5]. The main challenges consist of the unavailability or degradation of GNSS signals, the complexity of indoor environments, and the necessity of using low-grade devices [6]. Under such a circumstance, various positioning techniques have been researched, such as IEEE 802.11 WLAN (WiFi) [7,8,9,10], Radio Frequency Identification (RFID) [11], ultrasound [12], Ultra Wideband Beacons (UWB) [13], Bluetooth [14], ZigBee [15], infrared [16], and pseudolites [17]. Sometimes, multiple signal types are utilized simultaneously [18,19]. Since WiFi access points (AP) are installed ubiquitously nowadays, the WiFi approach has been much favored in most cases. Furthermore, it is also suitable in terms of both cost and accessibility [1].

WiFi indoor navigation methods are essentially divided into two categories: trilateration and fingerprinting [2]. In the trilateration approach, a radio propagation model is established and distance is estimated through the propagation model and the received signal strength (RSS) between WiFi access points (AP) and mobile stations [1]. In order to locate a mobile user, at least three APs have to be seen [8]. In the fingerprinting approach, there are usually two phases: offline and online phases. In the offline phase, the measurements of RSS at a number of selected reference points (RP) are stored with the position coordinates of the RPs [9]. As a result, a database is founded. In the online phase, the mobile user (MU) measures the RSS at the positioning point. Then the measurements are compared with the data in the database using an appropriate matching algorithm and the position estimation is obtained [20]. Compared with trilateration, fingerprinting gives better results and avoids complex modeling of signal propagation [8]. Therefore, WiFi fingerprinting method has gained much attention [6]. In this paper, a novel WiFi fingerprinting approach is proposed to achieve a better performance than is standard.

As Micro-Electro-Mechanical Systems (MEMS) develop rapidly, producing chip-based sensors is no longer a conundrum. Inertial sensors including accelerometers and gyroscopes are one of those devices. Since MEMS inertial sensors are small size, light weight, low cost and power saving, they have been widely used in positioning and navigation applications nowadays [21]. For consumer portable devices, dead-reckoning (DR) is commonly the algorithm applied to positioning with inertial sensors [6]. There are two categories of DR algorithms: inertial navigation system (INS) mechanism and pedestrian dead-reckoning (PDR). INS uses inertial sensors that measure inertial forces or rates, performs navigation mechanization and provides user with necessary navigation parameters [22]. INS is a source-independent positioning system [23] and is typically implemented for the inertial navigation [24]. The shortcoming is that inertial sensors provide only short-term accuracy and suffer from accuracy degradation over time as there exist sensor errors [22]. Although some deterministic sensor errors can be removed through calibration [25], low-cost MEMS inertial sensors suffer from significant run-to-run biases and thermal drifts [26]. In order to improve the navigation performance for pedestrians, PDR is developed to reduce the accumulated errors [27]. PDR has four vital procedures: step detection, step or stride length estimation, heading estimation, and 2D position calculation [28]. It is the relative means of determining of a new position from a previous known position [6]. The defect lies in the fact that PDR does not consider the effect of the roll and pitch. In this paper, an integrated MEMS solution is proposed to combine the advantages of both algorithms.

For the integration of WiFi and MEMS solutions, the literature [29] uses the robust extended Kalman filter (EKF) to fuse the information from WiFi/MEMS sensors in a multi-floor environment, while the research [24] presents a pedestrian navigator based on tightly coupled integration of low-cost MEMS sensors and WiFi for handheld devices. Using the same data from WiFi/MEMS sensors, various information fusion structures may lead to different results [6]. This paper proposes a novel two-stage EKF structure to fuse the information from WiFi/MEMS sensors. The proposed WiFi/MEMS solution improves the positioning accuracy. In addition, it provides an accurate navigation solution even when WiFi APs are deployed sparsely, and its stability has been validated.

The rest of this paper is organized as follows. In Section 2, literature review is discussed. In Section 3, the system overview is presented. The WiFi solution and PDR solution are described in Section 4 and Section 5, respectively. Section 6 provides the EKF-based integration of WiFi and MEMS. Details of experiments and results are shown in Section 7. Section 8 makes the conclusion.

## 2. Literature Review

Various researches have been conducted regarding WiFi indoor navigation methods. In [30], Bisio et al. proposed a computational and energy efficient probabilistic fingerprinting procedure, suited to be employed over smartphone platforms. In [31], Bisio et al. presented a novel approach where the training data were obtained by means of finite-difference time-domain simulations of the electromagnetic propagation in the considered scenario. In [32], Du et al. proposed an Access Point-centred indoor positioning system that overcame common limitations presented in conventional positioning systems, such as an excessive involvement of Mobile Devices. In [33], Lim et al. established the theoretical base and developed a localization algorithm for building a zero-configuration and robust indoor localization and tracking system to support location-based network services and management.

For the integration of WiFi and MEMS solutions, there are a number of existed researches. Frank et al. [34] presented an indoor positioning system for pedestrians combining Wireless LAN fingerprinting with foot mounted inertial and magnetometer sensors. Xiao et al. [35] proposed a region-based fingerprinting approach for indoor positioning in WiFi wireless networks and a stochastic system model to track the target’s position with an inertial measurement unit (IMU) integrated with the WiFi tag. Evennou and Marx [36] presented an aided DR navigation structure and signal processing algorithms for self localization of an autonomous mobile device by fusing PDR and WiFi signal strength measurements. Schatzberg et al. [37] presented a highly accurate indoor positioning system which was based on a new WiFi technology (protocol) and on MEMS inertial sensors. Iwase and Shibasaki [38] proposed a solution to reduce the accumulative error of PDR carried out with only the low cost sensors and WiFi in smartphones by realizing cooperative positioning among multiple pedestrians. Li et al. [39] developed a navigation algorithm which fused the WiFi received signal strength indicator (RSSI) and smartphone inertial sensor measurements. Table 1 lists the positioning performances from current WiFi/MEMS integrated systems in literature [24,34,35,36,37,38,39].

## 3. System Overview

**Figure 1:**
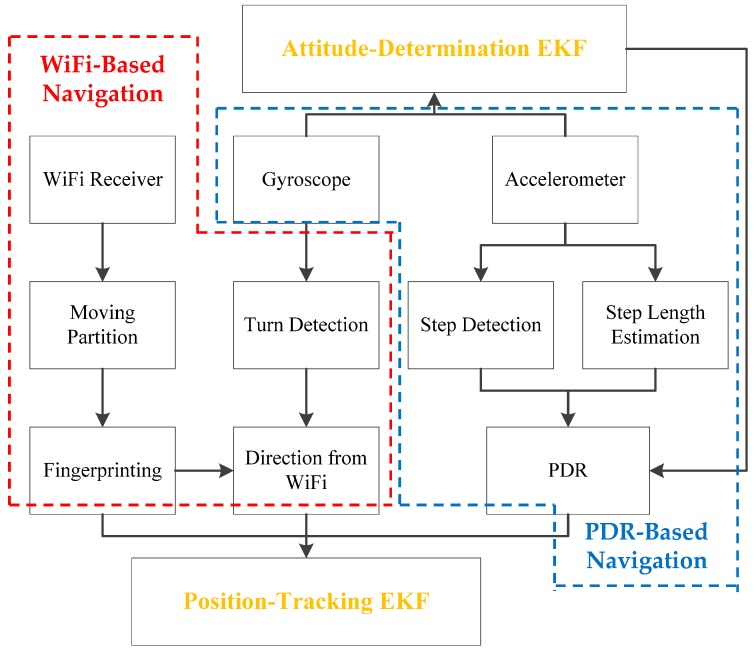
Block diagram.

The block diagram of the proposed EKF based WiFi/MEMS integration for indoor navigation is shown in Figure 1.

The proposed system mainly includes three modules: WiFi-based navigation, PDR-based navigation, and EKF-based WiFi/MEMS integration. In the presented WiFi solution, we pass RSS values to the fingerprinting scheme with “moving partition” to generate the MU position. Besides, if the turn detection fails, the MU direction measurement is obtained from WiFi positioning results. In PDR solution, the accelerometer data are utilized for step detection and step length estimation. Then PDR algorithm is applied to calculate the position of the MU. EKF-based integration includes an attitude-determination EKF and position-tracking EKF. In attitude-determination EKF, data from gyroscopes and accelerometers are used to generate the heading of the MU. In position-tracking EKF, results from WiFi and MEMS solutions are integrated to generate final navigation outcome.

## 4. WiFi-Based Navigation

### 4.1. Weighted K-Nearest Neighbors (WKNN) and Moving Partition

**Figure 2:**
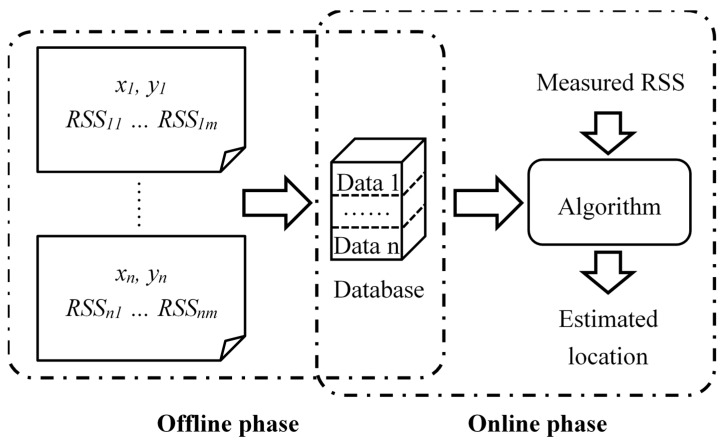
WiFi fingerprinting approach.

The whole process of WiFi fingerprinting is depicted in Figure 2. In fingerprinting scheme, to estimate the position of the MU, various matching algorithms have been proposed [8]. There are probabilistic methods [40,41], deterministic methods [42,43], and neural networks [44,45]. Among them, the Nearest Neighbor (NN) method is the most basic deterministic matching algorithm [9]. Firstly, the method calculates the distances between the measured RSS vector and the RSS vectors in the database. Then the position coordinates of the RP with the minimum distance is determined as that of the MU. Commonly, the distance is Euclidean distance [46], calculated by Equation (1). In other cases, Manhattan distance [2,47] and Mahalanobis distance [48] are used.

(1) $$D_{i}=\left\|S-M_{i}\right\|=\sqrt{\sum_{j=1}^{N}{\left|S_{j}-M_{i,j}\right|}^{2}}$$

where S and $M_{i}$ are the measured RSS vector and the *i*-th database fingerprint, respectively; $S_{j}$ and $M_{i,j}$ are the *j*-th elements in S and $M_{i}$, respectively; N is the number of available APs.

As the NN method has low stability and low accuracy, K-Nearest Neighbors (KNN) method has been developed [49], where *K* nearest neighbors (those with the shortest distance) are selected, and the arithmetic mean of position coordinates of the K RPs is regarded as estimate of the MU location [2], as shown in Equation (2).

(2) $$\left(\hat{x},\hat{y}\right)=\frac{1}{K}\sum_{i=1}^{K}\left(x_{i},y_{i}\right)$$

Furthermore, Weighted K-Nearest Neighbors (WKNN) method has been presented [50]. Instead of using the arithmetic mean, WKNN method utilizes the weighted mean of position coordinates of the *K* RPs to estimate the MU location. There are a number of approaches to determine weights [9]. In most cases, the multiplicative inverse of the distance calculated by Equation (1) determines the weight, and the MU is located by Equation (3).

(3) $$\left(\hat{x},\hat{y}\right)=\frac{\sum_{i=1}^{K}\frac{1}{D_{i}}\times \left(x_{i},y_{i}\right)}{\sum_{i=1}^{K}\frac{1}{D_{i}}}$$

where $D_{i}$ is the Euclidean distance calculated by Equation (1).

**Figure 3:**
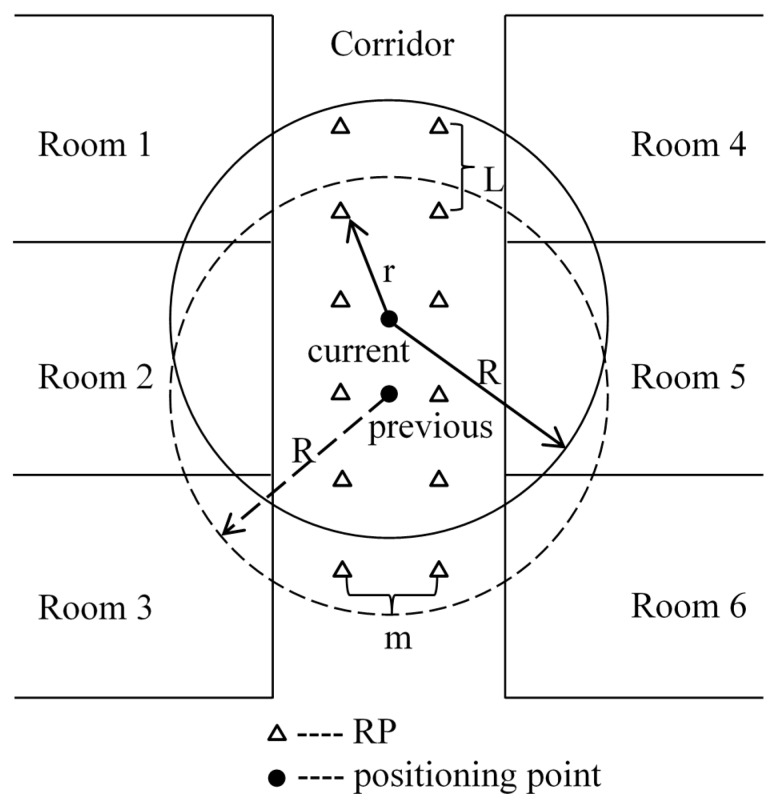
Moving partition.

The WKNN method takes full account of the effect of the current RPs for the estimated point. As a result, it performs better than KNN and NN methods [9]. However, it needs to search the overall space and go through the whole database fingerprints. For a big place and a huge database, the WKNN method seems to have low efficiency and the computation time can be unacceptable. In the current study, a partition solution is proposed and called “moving partition”. As shown in Figure 3, a circle located at the previous point is drawn with the given radius, and a partition in the circle is formed. The given radius is determined by the maximum moving distance of human in 1 s and can be set as 2.5 m. Suppose there are 10 RPs in the circle. These RPs are stored in an array. The current point is calculated within the scope of the 10 RPs using the WKNN method. Once the current point is located, a new partition is got with the same radius as the old one. The method to build the new partition is removing the last RPs in the array and putting in the new ones. The number of the changing RPs is determined by Equation (4).

(4) $$n=\left[\frac{m\left|R-r\right|}{L}\right]$$

where [ ] means the function of rounding down to integer; *L* is the distance between adjacent RPs; *m* is the number of RPs in a row; *R* is the radius of the partition circle; *r* is the distance between the current point and the RP at the edge of the circle in the forward direction. If R≥r, the partition moves forward. If R<r, it moves backward. Then the next point is calculated within the scope of RPs in the new partition.

With “moving partition”, the area of search space is greatly reduced. In addition, the work to update the partition is greatly reduced as well. Therefore, the computation time decreases significantly. The larger the test space is, the more obvious the effect is. In all, “moving partition” has the advantages of high efficiency and short computation time.

### 4.2. Direction from WiFi and Turn Detection

**Figure 4:**
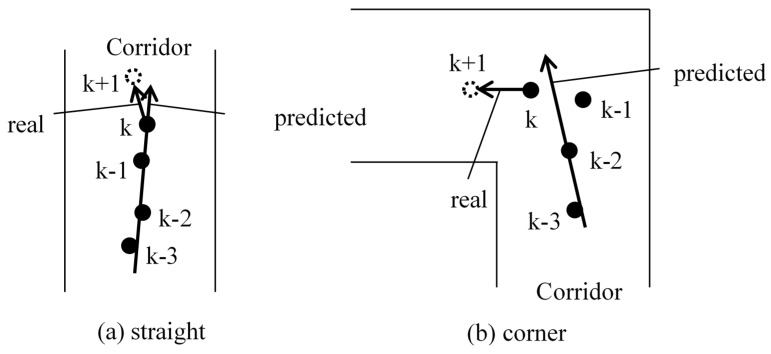
WiFi direction estimate. (**a**) Straight; (**b**) corner.

In the current study, the direction measurement of the MU is estimated utilizing the information from WiFi positioning results. As shown in Figure 4a, a line is drawn using the method of least squares with the information of 4 latest positioning points. If the coordinate of the kth point is larger than that of the (k-3)th point, the MU is moving forward, and vice versa. Since the MU walks straight most of the time, this method works in most cases. However, it becomes invalid at corner, as shown in Figure 4b. Therefore, a “turn detection” is necessary before the direction estimation. The detailed illustration is as follows.

**Figure 5:**
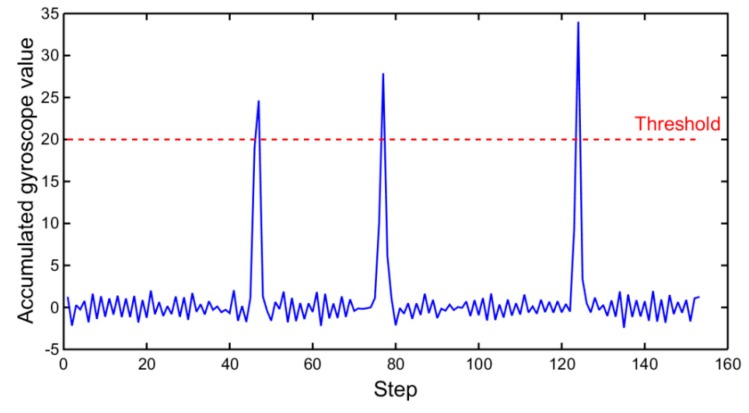
Accumulated gyroscope values during each step.

**Figure 6:**
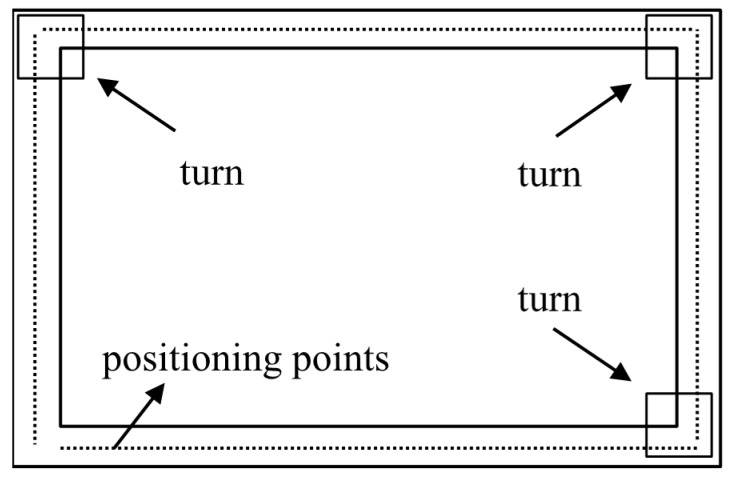
Turn detection.

In the current study, the turn detection is utilized to guarantee the effectiveness of direction from WiFi, and it can be achieved with gyroscopes. When the step detection is accomplished, the vertical angular velocities obtained from gyroscope data are accumulated during the period of each step. The accumulated values are depicted in Figure 5. As shown, there are three singular points. Combining Figure 6, it can be seen that the three singular points represent the three turns of the MU during walking. The vertical angular velocities vary intensively in short time at corners. Therefore, the accumulated gyroscope values can be used to detect turns. To achieve this, a threshold can be set, and once an accumulated value exceeds the threshold, a turn is detected. The turn detection only concerns the range of turn and is independent of accumulated errors of gyroscope data. In addition, it is important to note that the mobile phone must be kept as horizontal as possible during the detection [51]. In the current study, this requirement is satisfied.

## 5. Pedestrian Dead-Reckoning (PDR)-Based Navigation

### 5.1. Step Detection

Methods of step detection include peak detection, zero crossings, auto-correlation or template matching, and spectral analysis [52]. In the current study, peak detection is utilized. Firstly, the total acceleration can be calculated by

(5) $$a_{i}=\sqrt{a_{ix}^{2}+a_{iy}^{2}+a_{iz}^{2}}\;i=1,2,\ldots ,L$$

where $a_{ix},a_{iy},a_{iz}$ are accelerations observed in the carrier coordinate system and L is the sampling number. Then the local gravitational acceleration can be obtained by

(6) $$g=\frac{1}{M}\sum_{i=1}^{M}a_{i}^{s}$$

where $a_{i}^{s}$ is the acceleration observed when the mobile phone is kept static and M is the corresponding sampling number. Next, the gravitational acceleration is eliminated and the smoothed value is calculated by

(7) $$a_{i}^{\prime }=\frac{1}{2N+1}\sum_{j=i-N}^{i+N}\left(a_{j}-g\right)$$

where (2N+1) is the length of sliding window. Through sliding window and averaging, the high-frequency noise in acceleration signal can be restrained effectively. In addition, multi-peak waveform caused by body shake can be smoothed to single-peak waveform, which makes it easy to detect peaks [53].

**Figure 7:**
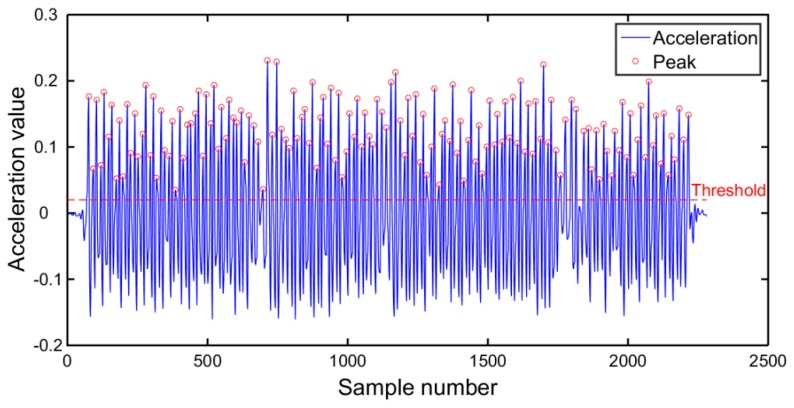
Peak detection.

The smoothed acceleration values calculated by Equation (7) are utilized to detect peaks. When detecting peaks, the following conditions must be obeyed. Firstly, the acceleration peak must be larger than a predetermined threshold. Secondly, the time interval between two adjacent peaks must be larger than a time threshold. The result of peak detection is illustrated in Figure 7, where red circles represent peaks detected.

### 5.2. Step Length Estimation

Step length estimation is used to estimate the moving distance of the pedestrian at each step [24]. In the current study, the practical model presented in [54] is utilized to estimate step length. This model assumes that the step length is proportional to the vertical movement of the human hip. The largest difference of the vertical acceleration at each step is used to calculate vertical movement [24]. The step length is estimated as follows.

(8) $$S_{acc}=\sqrt[{4}]{a_{z\max}-a_{z\min}}\cdot K$$

where $a_{z\max}$ and $a_{z\min}$ are the maximum and minimum values of the vertical acceleration, respectively; *K* is a calibrated constant parameter. This technique has been shown to measure distance walked to within ±8% across a variety of subjects of different leg lengths [54]. In the current study, the value of *K* is obtained through training, as shown in Table 2.

## 6. Extended Kalman Filter (EKF)-Based Integration

The EKF is usually utilized to fuse other information to reduce the drift of MEMS-based navigation approach [24]. It is a generalization of Kalman filter for nonlinear systems presenting small or moderate nonlinearities [55]. In this paper, a novel two-stage EKF structure to fuse the information from WiFi/MEMS sensors is proposed. The proposed structure contains attitude-determination EKF and position-tracking EKF, where the calculation of the former is used in the latter to generate final navigation results. The two-stage EKF structure has the advantage of high navigation accuracy. One thing to note is that all formulas in this section apply the International System of Units.

### 6.1. Attitude-Determination EKF

In the first stage, the information from gyroscopes and accelerometers is fused using the attitude-determination EKF to calculate the Euler angles between the carrier coordinate system and the navigation coordinate system. In the attitude-determination EKF, the system model is constructed utilizing the Euler angle differential equation and the measurement model is the conversion of tri-axial accelerations between the carrier coordinate system and the navigation coordinate system.

The state vector is written as:

(9) $$D_{k}={\left[\psi_{k}\;\theta_{k}\;\gamma_{k}\right]}^{T}$$

where ψ is the heading angle, θ is the pitching angle, γ is the roll angle, and ${}^{T}$ denotes transpose.

The initial state vector is set at:

(10) $${\hat{D}}_{0}={\left[0.5\pi \;0\;0\right]}^{T}$$

The initial covariance matrix of the state vector is set at:

(11) $$P_{10}=diag\left(\left[\begin{array}{ccc} 1 & 1 & 1 \end{array}\right]\right)$$

The system model is as follows:

(12) $$\{\begin{array}{c} \psi_{k}=\psi_{k-1}+\left(\frac{\sin\gamma_{k-1}}{\cos\theta_{k-1}}\omega_{x,k-1}-\frac{\cos\gamma_{k-1}}{\cos\theta_{k-1}}\omega_{z,k-1}\right)\Delta t+w_{\psi ,k-1}\\ \theta_{k}=\theta_{k-1}+\left(\cos\gamma_{k-1}\omega_{x,k-1}+\sin\gamma_{k-1}\omega_{z,k-1}\right)\Delta t+w_{\theta ,k-1}\\ \gamma_{k}=\gamma_{k-1}+\left(\sin\gamma_{k-1}\tan\theta_{k-1}\omega_{x,k-1}+\omega_{y,k-1}-\cos\gamma_{k-1}\tan\theta_{k-1}\omega_{z,k-1}\right)\Delta t+w_{\gamma ,k-1} \end{array}$$

where $\omega_{x},\omega_{y},\omega_{z}$ are angular velocities observed in the carrier coordinate system, $w_{\psi },w_{\theta },w_{\gamma }$ are noise, and Δt is the sampling interval.

The covariance matrix of system noise is set at:

(13) $$Q_{1}=diag\left(\left[\begin{array}{ccc} 1 & 1 & 1 \end{array}\right]\right)$$

The measurement vector is written as:

(14) $$A_{k}={\left[a_{x,k}\;a_{y,k}\;a_{z,k}\right]}^{T}$$

where $a_{x},a_{y},a_{z}$ are accelerations observed in the carrier coordinate system.

The measurement model is as follows:

(15) $$A_{k}=T_{k}^{-1}{\left[0\;0\;1\right]}^{T}+v_{k}$$

where $T_{k}$ is the direction cosine matrix calculated by Equation (16), and $v_{k}$ is noise.

(16) $$T_{k}=\left[\begin{array}{ccc} \cos\psi_{k} & -\sin\psi_{k} & 0\\ \sin\psi_{k} & \cos\psi_{k} & 0\\ 0 & 0 & 1 \end{array}\right]\left[\begin{array}{ccc} \cos\theta_{k} & 0 & \sin\theta_{k}\\ 0 & 1 & 0\\ -\sin\theta_{k} & 0 & \cos\theta_{k} \end{array}\right]\left[\begin{array}{ccc} 1 & 0 & 0\\ 0 & \cos\gamma_{k} & -\sin\gamma_{k}\\ 0 & \sin\gamma_{k} & \cos\gamma_{k} \end{array}\right]$$

The covariance matrix of measurement noise is set at:

(17) $$R_{1}=diag\left(\left[\begin{array}{ccc} 1 & 1 & 1 \end{array}\right]\right)$$

### 6.2. Position-Tracking EKF

In the second stage, the information from PDR and WiFi is fused using position-tracking EKF to calculate the MU position. In position-tracking EKF, the system model is established through PDR algorithm and the measurement model is the equivalence between WiFi results and PDR results. The heading angle obtained from attitude-determination EKF is utilized in position-tracking EKF.

The state vector is written as:

(18) $$B_{k}={\left[x_{k}\;y_{k}\;S_{k}\;\psi_{k}\right]}^{T}$$

where x,y are position coordinates of the MU, S is the step length, and ψ is the heading angle calculated by attitude-determination EKF.

The initial state vector is set at:

(19) $${\hat{B}}_{0}={\left[0.6\;0\;0.6\;0.5\pi \right]}^{T}$$

The initial covariance matrix of the state vector is set at:

(20) $$P_{20}=diag\left(\left[\begin{array}{cccc} 0.01 & 0.01 & 0.01 & 0.01 \end{array}\right]\right)$$

The system model is as follows:

(21) $$\{\begin{array}{c} x_{k}=x_{k-1}+S_{k-1}\cos\left(\pi -\psi_{k-1}\right)+w_{x,k-1}\\ y_{k}=y_{k-1}+S_{k-1}\sin\left(\pi -\psi_{k-1}\right)+w_{y,k-1}\\ S_{k}=S_{k-1}+w_{S,k-1}\\ \psi_{k}=\psi_{k-1}+\Delta \psi_{k-1}+w_{\psi ,k-1} \end{array}$$

where $w_{x},w_{y},w_{S},w_{\psi }$ are noise.

The covariance matrix of system noise is set at:

(22) $$Q_{2}=diag\left(\left[\begin{array}{cccc} 0.01 & 0.01 & 0.0001 & 0.01 \end{array}\right]\right)$$

The measurement vector is written as:

(23) $$C_{k}={\left[x_{WiFi,k}\;y_{WiFi,k}\;S_{acc,k}\;\psi_{WiFi,k}\right]}^{T}$$

where $x_{WiFi},y_{WiFi}$ are position coordinates from WiFi results, $S_{acc}$ is the step length calculated by Equation (8), and $\psi_{WiFi}$ is the heading angle from WiFi results.

The measurement model is as follows:

(24) $$C_{k}=B_{k}+u_{k}$$

where $B_{k}$ is the state vector and $u_{k}$ is noise.

The covariance matrix of measurement noise is set at:

(25) $$R_{2}=diag\left(\left[\begin{array}{cccc} 1000 & 1000 & 10 & 100 \end{array}\right]\right)$$

The two-stage EKF algorithm for WiFi/MEMS integration is summarized in Algorithm 1.

**Algorithm 1** Two-stage EKF for WiFi/MEMS integration.Step 1: Initialize the attitude-determination EKF${\hat{D}}_{0}={\left[0.5\pi \;0\;0\right]}^{T}$, $P_{10}=diag\left(\left[\begin{array}{ccc} 1 & 1 & 1 \end{array}\right]\right)$, k=1Step 2: Attitude-determination EKF$F_{k-1}={\left(\partial f_{k-1}/\partial D\right)|}_{{\hat{D}}_{k-1}^{+}}$, where $f_{k-1}\left(\cdot \right)$ is the system model defined in Equation (12)
$P_{1k}^{-}=F_{k-1}P_{1,k-1}^{+}F_{k-1}^{T}+Q_{1,k-1}$
${\hat{D}}_{k}^{-}=f_{k-1}\left({\hat{D}}_{k-1}^{+},u_{k-1},0\right)$, where $u_{k-1}={\left[\omega_{x,k-1}\;\omega_{y,k-1}\;\omega_{z,k-1}\right]}^{T}$
$H_{k}={\left(\partial T_{k}^{-1}/\partial D\right)|}_{{\hat{D}}_{k}^{-}}\times {\left[0\;0\;1\right]}^{T}$

$K_{1k}=P_{1k}^{-}H_{k}^{T}{\left(H_{k}P_{1k}^{-}H_{k}^{T}+R_{1k}\right)}^{-1}$

${\hat{D}}_{k}^{+}={\hat{D}}_{k}^{-}+K_{1k}\left(A_{k}-T_{k}^{-1}{\left[0\;0\;1\right]}^{T}\right)$
$P_{1k}^{+}=\left(I-K_{1k}H_{k}\right)P_{1k}^{-}$, where I is the identity matrixStep 3: k=k+1 and return to Step 2Step 4: Initialize the position-tracking EKF${\hat{B}}_{0}={\left[0.6\;0\;0.6\;0.5\pi \right]}^{T}$, $P_{20}=diag\left(\left[\begin{array}{cccc} 0.01 & 0.01 & 0.01 & 0.01 \end{array}\right]\right)$, k=1Step 5: Position-tracking EKF$G_{k-1}={\left(\partial g_{k-1}/\partial B\right)|}_{{\hat{B}}_{k-1}^{+}}$, where $g_{k-1}\left(\cdot \right)$ is the system model defined in Equation (21)
$P_{2k}^{-}=G_{k-1}P_{2,k-1}^{+}G_{k-1}^{T}+Q_{2,k-1}$

${\hat{B}}_{k}^{-}=g_{k-1}\left({\hat{B}}_{k-1}^{+},0\right)$

$K_{2k}=P_{2k}^{-}{\left(P_{2k}^{-}+R_{2k}\right)}^{-1}$

${\hat{B}}_{k}^{+}={\hat{B}}_{k}^{-}+K_{2k}\left(C_{k}-{\hat{B}}_{k}^{-}\right)$

$P_{2k}^{+}=\left(I-K_{2k}\right)P_{2k}^{-}$
Step 6: k=k+1 and return to Step 5

## 7. Experiment Description and Results

### 7.1. Experiment Description and Comparison of “Moving Partition” and WKNN

To evaluate the performance of the proposed indoor navigation method, several experiments were performed. The experiment platform can be any Android smartphone equipped with an accelerometer triad, a gyroscope triad and a WiFi receiver. In the current study, a Samsung Galaxy S2 and a Mi 5s were used. The IMU data was collected through the app “Physics Toolbox Suite”, and the WiFi data was collected through the app “RSS Collection”.

**Figure 8:**
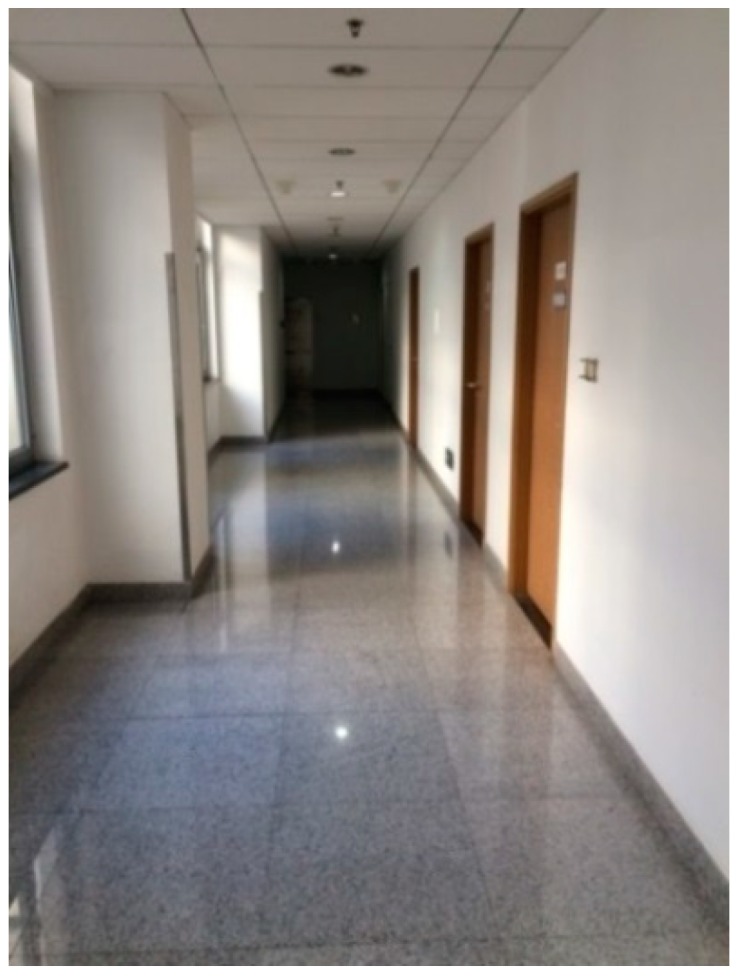
Test environment.

**Figure 9:**
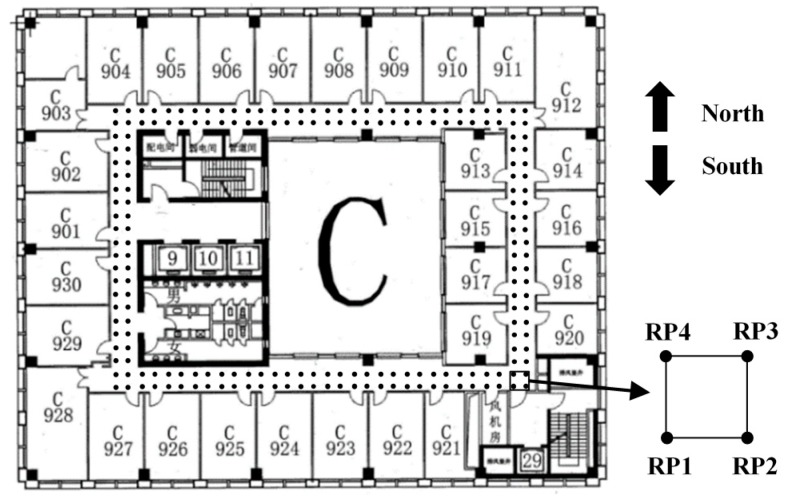
Floor layout.

The experiments were conducted in the ninth floor of Block C, New Main Building at Beihang University, as shown in Figure 8. The layout of the floor is shown in Figure 9. The size of test area is around 30 × 20 m^2^ and the loop corridor is 1.8 m wide. There were 158 RPs and 155 positioning points in all. A total of 270 APs were involved in testing. The exact positions of these APs are unknown. To eliminate the effect of randomness of human behavior [56], the offline training data were collected systematically using a 1.2-m grid in this study. At each of the 158 grid points, 40 observations were recorded, of which 10 were collected toward each direction of east, south, west and north. A five-order median filter was applied to the 40 observations, and then the mean value was calculated and stored in the database. In the online phase, the tester walked around the loop corridor and collected RSS values at each point. Then the RSS values received at each positioning point were sorted and the top 20 APs were selected. The position coordinate of the starting point is (0.6 m, 0) and the corresponding heading, pitching and roll angles are 0.5π, 0 and 0, respectively. Finally, a total of 155 points were recorded in one loop.

The performance comparison of “moving partition” and the traditional WKNN method is illustrated in Table 3. As shown, the running time of “moving partition” and WKNN on the same device was 0.28 s and 0.62 s, respectively. With “moving partition”, the computation time decreased by more than half. Besides, the average positioning error reduced from 1.9840 m to 1.8857 m. Therefore, “moving partition” enhances navigation performance.

### 7.2. Comparison of WiFi, MEMS, and WiFi/MEMS Integration

**Figure 10:**
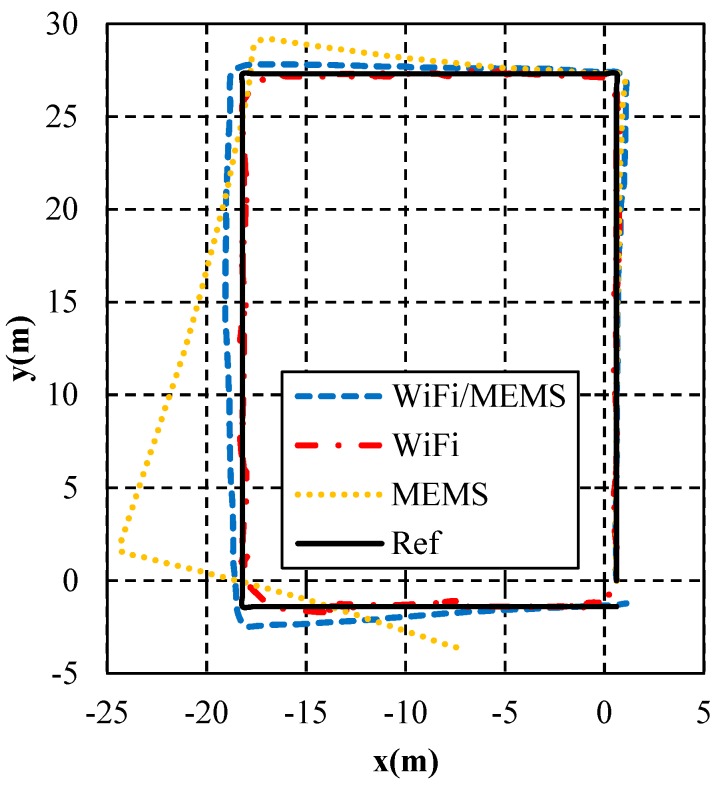
Positioning results.

Figure 10 depicts the navigation performance comparison of WiFi (with “moving partition”), MEMS, and WiFi/MEMS integration (with “moving partition”) in one loop. The “Ref” represents the actual trajectory which the tester walked along. As shown, the estimation of WiFi/MEMS integration proposed in this paper has a nice performance, making a good fit with the reference trajectory. Also, compared with WiFi, the phenomenon of reciprocating motion has been eliminated significantly. This figure depicts the estimation result of MEMS as well. As is known, the estimation error accumulates quickly for low grade MEMS sensors in smart devices which usually have large accelerometer and gyroscope biases. In the first part of the corridor, the MEMS estimation keeps good performance. However, when turning to the second part, the trajectory deviates. This phenomenon becomes more and more serious in the third and fourth parts of the corridor. Therefore, MEMS needs to be integrated with WiFi using the EKF to achieve a more accurate estimation, as illustrated in the above section. The result of WiFi/MEMS integration in Figure 10 has shown its advantage that it has the highest accuracy.

The detailed estimation errors and running time of these algorithms are listed in Table 4. As shown, the maximum error of WiFi/MEMS is 1.2498 m, which is 16.07% of MEMS and 23.97% of WiFi. The mean error of WiFi/MEMS is 0.6835 m, which is 24.67% of MEMS and 36.25% of WiFi. The root mean square (RMS) of WiFi/MEMS position errors is 0.7926 m, which is 20.59% of MEMS and 36.60% of WiFi. Therefore, the EKF-based WiFi/MEMS algorithm proposed in this paper has improved the estimation accuracy. Besides, it has low computational complexity as the running time is short.

**Figure 11:**
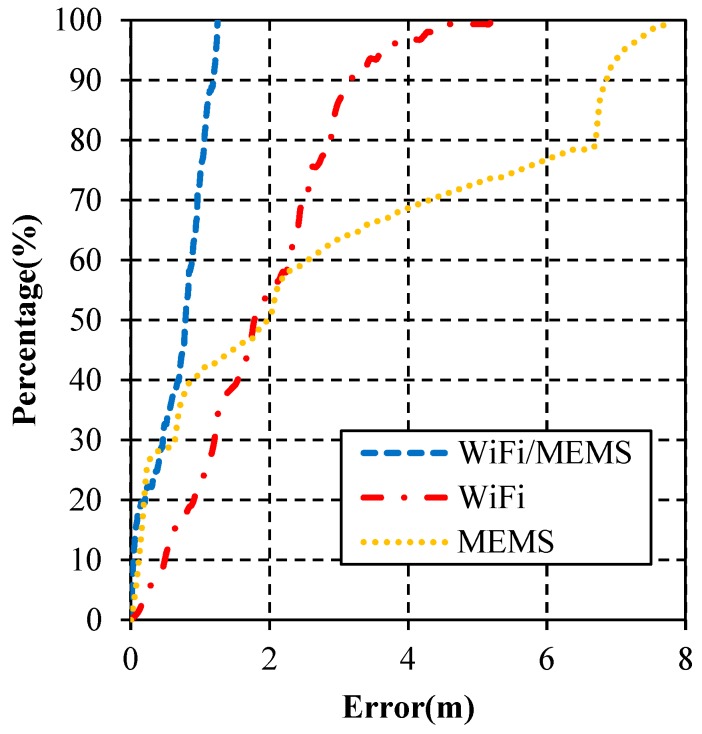
Cumulative error percentages.

Furthermore, the error probabilities of these algorithms are depicted in Figure 11. As shown, the maximum error of WiFi/MEMS is far smaller than the other two. The cumulative error percentages of MEMS and WiFi/MEMS are close when the error is smaller than 0.5 m, but WiFi/MEMS solution quickly achieves the best performance when the error is larger than its mean error, showing a very stable estimation. In all, this figure illustrates the advantage of EKF-based WiFi/MEMS integration algorithm as well.

### 7.3. Results Using Different Numbers of Access Points (APs)

To further validate the navigation performance of the proposed WiFi/MEMS solution, estimation results using different numbers of APs and in different loops are discussed as follows.

**Figure 12:**
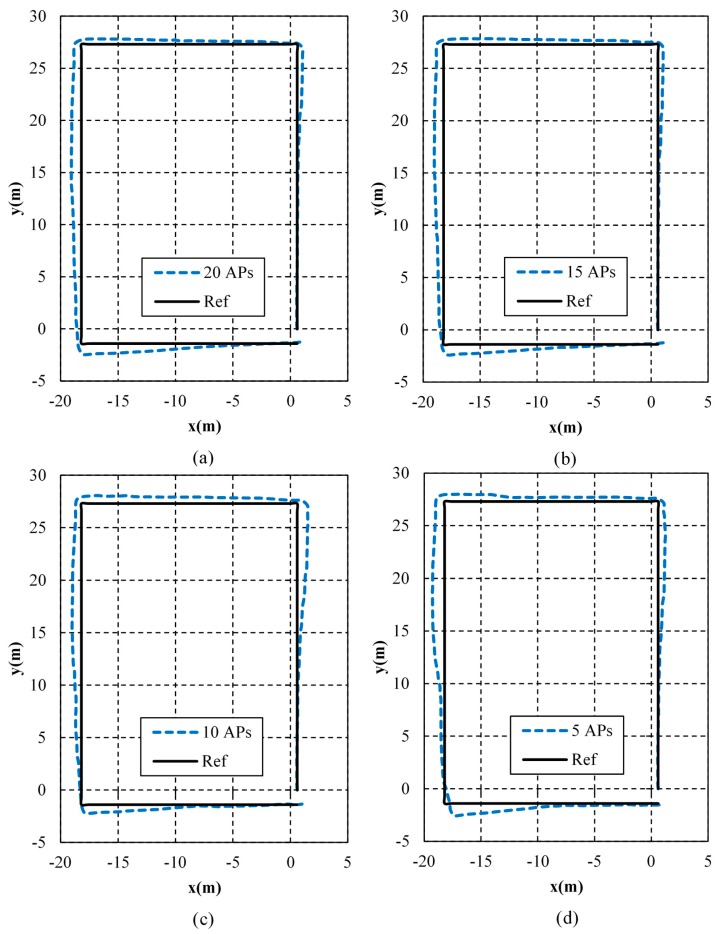
Positioning results using different numbers of APs. (**a**) 20 APs; (**b**) 15 APs; (**c**) 10 APs; (**d**) 5 APs.

As shown in Figure 12, the algorithms using 20 APs, 15 APs, 10 APs and 5 APs are compared. In this figure, the results of 20 APs and 15 APs are very close. The worst is the estimation using 5 APs. However, it still keeps good performance in most areas, illustrating that the WiFi/MEMS solution still works well when there are very few APs available.

The detailed estimation errors using different numbers of APs are listed in Table 5. As shown, the maximum error is the smallest when using 10 APs, while the mean error and the RMS of position errors are the smallest when using 15 APs. Therefore, the result of 15 APs fits the best. The mean error when using 15 APs is 0.6823 m, which is 82.66% of 5 APs, 94.41% of 10 APs, and 99.82% of 20 APs. The RMS of position errors using 15 APs is 0.7868 m, which is 84.12% of 5 APs, 99.27% of 20 APs, and 99.42% of 10 APs. As a result, more APs do not necessarily mean more accurate estimation.

**Figure 13:**
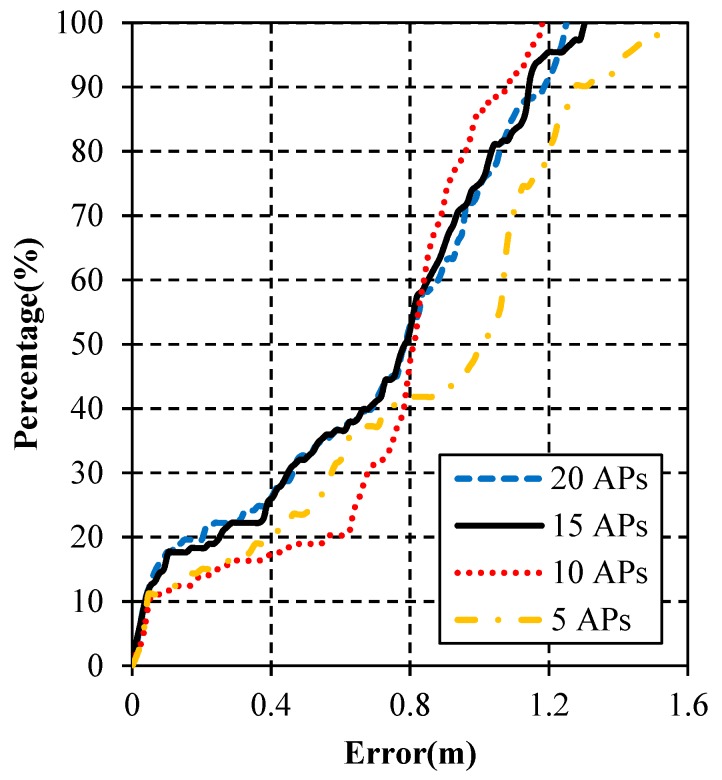
Cumulative error percentages using different numbers of APs.

The error probabilities using different numbers of APs are depicted in Figure 13. As shown, there is no big difference between the results of 20 APs and 15 APs. Obvious difference appears when the results of 10 APs and 5 APs are considered. In all, the positioning result using 15 APs has the best navigation performance and the result using 5 APs is the worst.

### 7.4. Results in Different Loops

**Figure 14:**
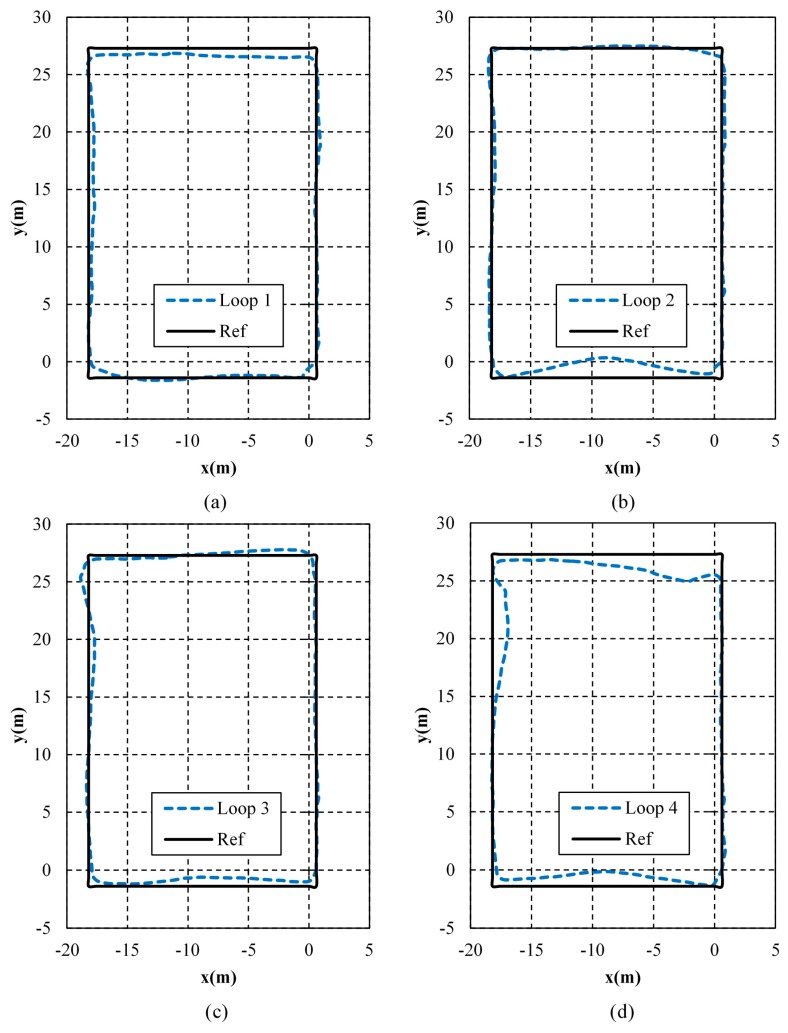
Positioning results in different loops. (**a**) Loop 1; (**b**) Loop 2; (**c**) Loop 3; (**d**) Loop 4.

To verify the stability of the proposed WiFi/MEMS solution, the tester walked along the loop corridor for four loops continuously. Also, a different smartphone was used to record the accelerations and angular velocities in order to prove the validity of the proposed WiFi/MEMS solution on various devices. The positioning results in different loops are shown in Figure 14. As shown, there is no obvious difference among the first three loops, and a little decrease of estimation accuracy occurs in the fourth loop. Since the proposed algorithm is the integration of WiFi and MEMS, it is inevitable that it is affected by the accumulated errors from MEMS. However, this algorithm can control these errors within a relatively lower level. Besides, further work should focus on how to compensate them at certain points.

The detailed estimation errors in different loops are listed in Table 6. As shown, the smallest of mean errors appears in Loop 2, while the maximum error and the RMS of errors are the smallest in Loop 3. The mean error in Loop 2 is 0.9895 m, which is 81.72% of Loop 4, 90.06% of Loop 1, and 99.85% of Loop 3. The maximum error in Loop 3 is 2.9834 m, which is 93.25% of Loop 4, 96.65% of Loop 1, and 98.26% of Loop 2. The RMS of position errors in Loop 3 is 1.1833 m, which is 83.76% of Loop 4, 94.03% of Loop 1, and 99.12% of Loop 2. As can be seen, the estimation of the proposed WiFi/MEMS solution keeps stable in the four loops.

**Figure 15:**
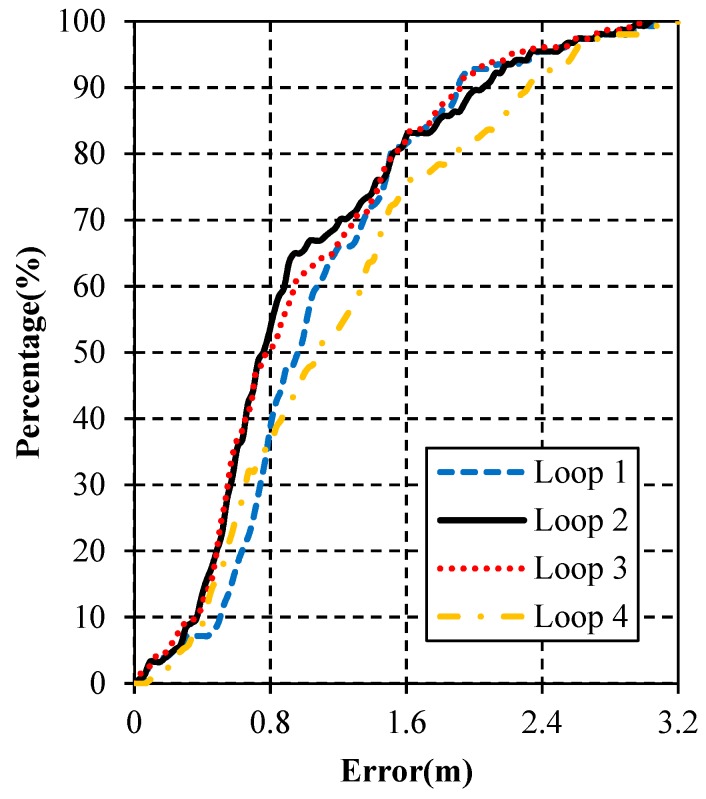
Cumulative error percentages in different loops.

The error probabilities in different loops are depicted in Figure 15. As shown, the order of the four loops varies in different intervals. Besides, the accuracy does not vary remarkably among the four loops. In all, the stability and validity on different devices of the proposed algorithm have been proven.

## 8. Conclusions

This paper presents a novel WiFi/MEMS fusion structure for indoor navigation. In the WiFi fingerprinting scheme, a partition solution called “moving partition” is originally proposed. With “moving partition”, the computation time decreases by more than half compared with the traditional WKNN method. Also, the direction measurement of the MU using the information of four latest positioning points from WiFi is presented, and a “turn detection” is applied before the direction fusion. Then a novel two-stage structure to integrate the information from WiFi/MEMS sensors using the EKF is proposed. In the first stage, the information from the gyroscope and the accelerometer is fused using attitude-determination EKF to calculate the Euler angles. In the second stage, the information from PDR and WiFi is fused using position-tracking EKF to calculate the MU position.

With these improvements, the navigation performance of the proposed WiFi/MEMS integration solution is compared with results from WiFi and MEMS. Experiment results show that the RMS position error of the proposed WiFi/MEMS solution is 0.7926 m, which is 20.59% of MEMS and 36.60% of WiFi. Furthermore, the presented WiFi/MEMS algorithm still works well when there are very few APs available and its stability has been proven. Therefore, the proposed WiFi/MEMS solution has been validated in indoor tests, and its performance is illustrated to be very competitive for indoor navigation. Furthermore, it can be easily applied on most handheld devices, such as smartphones, and presents broad market prospect.

## Figures and Tables

**Table 1:** Positioning performances from other systems. IMU– inertial measurement unit; RMS–root mean square.

System	Algorithm	Accuracy
[24]	WiFi propagation model + Smartphone IMU	RMS: 3.47 m
[34]	WiFi fingerprinting + Shoe IMU	RMS: 1.65 m
[35]	WiFi fingerprinting + Trolley IMU	Mean: around 2.00 m
[36]	WiFi fingerprinting + Shoe IMU	Mean: 1.53 m
[37]	WiFi ToF + Strapped IMU	Max: 2.50 m
[38]	WiFi propagation model + Smartphone IMU + multi-person collaborative positioning	RMS: around 5.00 m
[39]	WiFi propagation model + Smartphone IMU	Mean: 7.83 m

**Table 2:** *K* value and training parameters.

Distance (m)	Step Counts	Step Length (m)	*K*
25.8	43	0.6	0.861

**Table 3:** Performance comparison of “moving partition” and Weighted K-Nearest Neighbors (WKNN).

	Moving Partition	WKNN
running time (s)	0.28	0.62
average positioning error (m)	1.8857	1.9840

**Table 4:** Positioning errors and running time.

	Maximum (m)	Mean (m)	RMS (m)	Running Time (s)
WiFi	5.2147	1.8857	2.1657	0.28
MEMS	7.7754	2.7703	3.8502	0.81
WiFi/MEMS	1.2498	0.6835	0.7926	1.20

**Table 5:** Positioning errors using different numbers of APs.

	Maximum (m)	Mean (m)	RMS (m)
20 APs	1.2498	0.6835	0.7926
15 APs	1.3027	0.6823	0.7868
10 APs	1.1806	0.7227	0.7914
5 APs	1.5388	0.8254	0.9353

**Table 6:** Positioning errors in different loops.

	Maximum (m)	Mean (m)	RMS (m)
Loop 1	3.0867	1.0987	1.2584
Loop 2	3.0363	0.9895	1.1938
Loop 3	2.9834	0.9910	1.1833
Loop 4	3.1994	1.2109	1.4127

## References

[1] Shin B., Lee J.H., Lee T., Kim H.S. (2012). Enhanced weighted K-nearest neighbor algorithm for indoor Wi-Fi positioning systems. Int. J. Netw. Comput. Adv. Inf. Manag..

[2] Li B., Wang Y., Lee H.K., Dempster A., Rizos C. (2005). Method for yielding a database of location fingerprints in WLAN. IEE Proc.-Commun..

[3] Rahim A. (2012). Heading Drift Mitigation for Low-Cost Inertial Pedestrian Navigation. Ph.D. Dissertation.

[4] Li X., Wang J., Li T. (2013). Seamless positioning and navigation by using geo-referenced images and multi-sensor data. Sensors.

[5] Yassin A., Nasser Y., Awad M., Al-Dubai A., Liu R., Yuen C., Raulefs R., Aboutanios E. (2017). Recent advances in indoor localization: A survey on theoretical approaches and applications. IEEE Commun. Surv. Tutor.

[6] Li Y., Zhuang Y., Zhang P., Lan H., Niu X., El-Sheimy N. (2016). An improved inertial/wifi/magnetic fusion structure for indoor navigation. Inf. Fusion.

[7] Caso G., Nardis L.D. (2017). Virtual and oriented WiFi fingerprinting indoor positioning based on multi-wall multi-floor propagation models. Mob. Netw. Appl..

[8] Khodayari S., Maleki M., Hamedi E. A RSS-based fingerprinting method for positioning based on historical data. Proceedings of the International Symposium on Performance Evaluation of Computer and Telecommunication Systems.

[9] Wang B., Zhao Y., Zhang T., Hei X. An improved integrated fingerprint location algorithm based on WKNN. Proceedings of the 2017 29th Chinese Control and Decision Conference (CCDC).

[10] Tuta J., Juric M.B. (2016). A self-adaptive model-based Wi-Fi indoor localization method. Sensors.

[11] Seol S., Lee E.K., Kim W. (2017). Indoor mobile object tracking using RFID. Future Gener. Comput. Syst..

[12] Lindo A., García E., Ureña J., Pérez M.C., Hernández A. (2015). Multiband waveform design for an ultrasonic indoor positioning system. IEEE Sens. J..

[13] Chen P., Kuang Y., Chen X. (2017). A UWB/improved PDR integration algorithm applied to dynamic indoor positioning for pedestrians. Sensors.

[14] Zhou C., Yuan J., Liu H., Qiu J. (2017). Bluetooth indoor positioning based on RSSI and Kalman filter. Wirel. Pers. Commun..

[15] Aykaç M., Erçelebi E., Aldin N.B. (2017). ZigBee-based indoor localization system with the personal dynamic positioning method and modified particle filter estimation. Analog Integr. Circ. Sig. Process..

[16] Wang K., Nirmalathas A., Lim C., Alameh K., Li H., Skafidas E. (2017). Indoor infrared optical wireless localization system with background light power estimation capability. Opt. Express.

[17] Li X., Zhang P., Guo J., Wang J., Qiu W. (2017). A new method for single-epoch ambiguity resolution with indoor pseudolite positioning. Sensors.

[18] Tuta J., Juric M.B. (2018). MFAM: Multiple frequency adaptive model-based indoor localization method. Sensors.

[19] Liu R., Yuen C., Do T.-N., Tan U.-X. (2017). Fusing similarity-based sequence and dead reckoning for indoor positioning without training. IEEE Sens. J..

[20] Hu J., Liu D., Yan Z., Liu H. (2019). Experimental analysis on weight K-nearest neighbor indoor fingerprint positioning. IEEE Internet Things.

[21] Shaeffer D.K. (2013). MEMS inertial sensors: A tutorial overview. IEEE Commun. Mag..

[22] Titterton D.H., Weston J.L. (2004). Strapdown Inertial Navigation Technology.

[23] Niu X., Ban Y., Zhang Q., Zhang T., Zhang H., Liu J. (2015). Quantitative analysis to the impacts of IMU quality in GPS/INS deep integration. Micromachines.

[24] Zhuang Y., El-Sheimy N. (2016). Tightly-coupled integration of WiFi and MEMS sensors on handheld devices for indoor pedestrian navigation. IEEE Sens. J..

[25] Bonnet S., Bassompierre C., Godin C., Lesecq S., Barraud A. (2009). Calibration methods for inertial and magnetic sensors. Sens. Actuators A Phys..

[26] Niu X., Li Y., Zhang H., Wang Q., Ban Y. (2013). Fast thermal calibration of low-grade inertial sensors and inertial measurement units. Sensors.

[27] Yu J., Na Z., Liu X., Deng Z. (2019). WiFi/PDR-integrated indoor localization using unconstrained smartphones. EURASIP J. Wirel. Commun..

[28] Zhuang Y., Lan H., Li Y., El-Sheimy N. (2015). PDR/INS/WiFi integration based on handheld devices for indoor pedestrian navigation. Micromachines.

[29] Tian Z., Fang X., Zhou M., Li L. (2015). Smartphone-based indoor integrated WiFi/MEMS positioning algorithm in a multi-floor environment. Micromachines.

[30] Bisio I., Lavagetto F., Marchese M., Sciarrone A. (2016). Smart probabilistic fingerprinting for WiFi-based indoor positioning with mobile devices. Pervasive Mob. Comput..

[31] Bisio I., Cerruti M., Lavagetto F., Marchese M., Pastorino M., Randazzo A., Sciarrone A. (2014). A trainingless WiFi fingerprint positioning approach over mobile devices. IEEE Antenn Wirel. Propag..

[32] Du X., Yang K., Bisio I., Lavagetto F., Sciarrone A. An AP-centred smart probabilistic fingerprint system for indoor positioning. Proceedings of the 2018 IEEE International Conference on Communications (ICC).

[33] Lim H., Kung L.-C., Hou J.C., Luo H. (2010). Zero-configuration indoor localization over IEEE 802.11 wireless infrastructure. Wirel. Netw..

[34] Frank K., Krach B., Catterall N., Robertson P. Development and evaluation of a combined WLAN and inertial indoor pedestrian positioning system. Proceedings of the 4th ION GNSS.

[35] Xiao W., Ni W., Yue K.T. Integrated Wi-Fi fingerprinting and inertial sensing for indoor positioning. Proceedings of the 2011 International Conference on Indoor Positioning and Indoor Navigation (IPIN).

[36] Evennou F., Marx F. (2006). Advanced integration of WiFi and inertial navigation systems for indoor mobile positioning. EURASIP J. Appl. Signal. Process..

[37] Schatzberg U., Banin L., Amizur Y. Enhanced WiFi ToF indoor positioning system with MEMS-based INS and pedometric information. Proceedings of the IEEE/ION Position, Location and Navigation Symposium (PLANS).

[38] Iwase T., Shibasaki R. Infra-free indoor positioning using only smartphone sensors. Proceedings of the 2013 International Conference on Indoor Positioning and Indoor Navigation (IPIN).

[39] Li W.W., Iltis R.A., Win M.Z. A smartphone localization algorithm using RSSI and inertial sensor measurement fusion. Proceedings of the IEEE Global Communications Conference (GLOBECOM).

[40] Liang W., Wang Y., Wu Z., Mao B., Cao J. (2018). Indoor region localization with asynchronous sensing data: A Bayesian probabilistic model. IEEE Sens. J..

[41] Mirowski P., Milioris D., Whiting P., Ho T.K. (2014). Probabilistic radio-frequency fingerprinting and localization on the run. Bell Labs Tech. J..

[42] Tayebi A., Gomez J., Saez de Adana F., Gutierrez O. (2009). The application of ray-tracing to mobile localization using the direction of arrival and received signal strength in multipath indoor environments. Prog. Electromagn. Res..

[43] Khordad M., Maleki M. K nearest neighbors based on lateration for WLAN location estimation. Proceedings of the IEEE, IET International Symposium on Communication Systems, Networks & Digital Signal Processing (CSNDSP).

[44] Li Y., Gao Z., He Z., Zhuang Y., Radi A., Chen R., El-Sheimy N. (2019). Wireless fingerprinting uncertainty prediction based on machine learning. Sensors.

[45] Akram B.A., Akbar A.H., Shafiq O. (2018). HybLoc: Hybrid indoor Wi-Fi localization using soft clustering-based random decision forest ensembles. IEEE Access.

[46] Wang Y., Xiu C., Zhang X., Yang D. (2018). WiFi indoor localization with CSI fingerprinting-based random forest. Sensors.

[47] Li C., Qiu Z., Liu C. (2017). An improved weighted K-nearest neighbor algorithm for indoor positioning. Wireless Pers. Commun..

[48] Zhou J., Yeung W.M., Ng J.K. Enhancing indoor positioning accuracy by utilizing signals from both the mobile phone network and the wireless local area network. Proceedings of the 22nd International Conference on Advanced Information Networking and Applications.

[49] Xu H., Ding Y., Li P., Wang R., Li Y. (2017). An RFID indoor positioning algorithm based on Bayesian probability and K-nearest neighbor. Sensors.

[50] Fang X., Jiang Z., Nan L., Chen L. (2018). Optimal weighted K-nearest neighbour algorithm for wireless sensor network fingerprint localisation in noisy environment. IET Commun..

[51] Liu C. (2015). Study on the Pedestrian Indoor Positioning Algorithms Based on WIFI and Inertial Technology. M.S. Thesis.

[52] Harle R. (2013). A survey of indoor inertial positioning systems for pedestrians. IEEE Commun. Surv. Tutor..

[53] Chen W. (2010). Research on GPS/Self-Contained Sensors Based Seamless Outdoor/Indoor Pedestrian Positioning Algorithm. Ph.D. Dissertation.

[54] Weinberg H. (2002). Using the ADXL202 in pedometer and personal navigation applications. Application Note AN-602.

[55] Santos-Ruiz I., Bermudez J.R., Lopez-Estrada F.R., Puig V., Torres L., Delgado-Aguinaga J.A. (2018). Online leak diagnosis in pipelines using an EKF-based and steady-state mixed approach. Control Eng. Pract..

[56] Roos T., Myllymäki P., Tirri H., Misikangas P., Sievänen J. (2002). A probabilistic approach to WLAN user location estimation. Int. J. Wirel. Inf. Netw..

